# Impact of body mass index on perioperative and oncological outcomes in elderly patients undergoing minimally invasive McKeown esophagectomy for esophageal squamous cell carcinoma

**DOI:** 10.1002/cam4.4660

**Published:** 2022-03-21

**Authors:** Chaoyang Tong, Huijie Lu, Hongwei Zhu, Jingxiang Wu

**Affiliations:** ^1^ Department of Anesthesiology, Shanghai Chest Hospital Shanghai Jiao Tong University Shanghai China; ^2^ Department of Anesthesiology, Shanghai Children's Medical Center, School of Medicine Shanghai Jiao Tong University Shanghai China

**Keywords:** elderly, McKeown, minimally invasive esophagectomy, outcomes, SCC

## Abstract

**Background:**

The association between elevated body mass index (BMI) and perioperative and oncological outcomes among elderly patients undergoing minimally invasive McKeown esophagectomy (MIE) remains unclear.

**Methods:**

We performed a single‐center retrospective analysis of 526 consecutive patients aged 65 years or older who underwent MIE for esophageal squamous cell carcinoma (SCC) between January 2016 and December 2019. Two groups were stratified by BMI: normal (18.5 ≤ BMI < 24 kg/m^2^) and elevated groups (BMI ≥ 24 kg/m^2^). A 1:1 propensity score matching (PSM) analysis was used to compare perioperative and oncological outcomes between the two groups.

**Results:**

A total of 480 elderly patients were eventually enrolled, with a mean age of 70.2 years (range: 65–87), and 185 patients were eligible for elevated BMI, with a mean BMI of 26.3 ± 1.9 kg/m^2^. Compared with the normal BMI group, the elevated BMI group had prolonged operation time (261.7 ± 57.2 vs. 278.9 ± 62.7 mins, *p* = 0.002) and increased incidence of intraoperative hypoxemia (12.2% vs. 21.6%, *p* = 0.006). The differences in intraoperative estimated blood loss, transfusion, new‐onset arrhythmia, and conversion rates and postoperative outcomes regarding pulmonary and surgical complications, intensive care unit and 30‐day readmissions, the length of hospital stay, and oncological outcomes regarding R0 dissection, and the number of dissected lymph nodes between two groups were comparable. After a 1:1 PSM analysis, there was no significant difference in both perioperative and oncological outcomes between two groups.

**Conclusions:**

Among elderly patients undergoing MIE for esophageal SCC, there was insufficient evidence to demonstrate that elevated BMI could increase perioperative and oncological adverse outcomes.

## Graphical abstract

After a 1:1 PSM analysis, there was insufficient evidence that elevated BMI increased perioperative and oncological outcomes among elderly patients undergoing MIE for esophageal SCC.

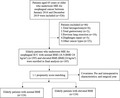

## 1 INTRODUCTION

In recent decades, the prevalence of overweight and obesity has shifted dramatically,^1^, ^2^ and metabolic diseases associated with elevated weight such as hypertension, diabetes mellitus, and hyperlipidemia have also increased gradually,^3^, ^4^, ^5^ leading to a worldwide health problem. In addition, with the aging of society, the special attributes of elderly surgical patients, such as frailty, cognitive decline, impaired preoperative lung function, and tissue fragility, make the effect of elevated weight on perioperative outcomes more complicated.^6^, ^7^, ^8^ Indeed, the rate of weight gain in older patients receiving esophageal cancer surgery has increased over the past few decades.^6^ And the ultimate goal of esophageal cancer surgery is to be accompanied by therapeutic resection to ensure short ‐ and long‐term prognosis.^9^, ^10^

However, the majority of patients undergoing radical resection of esophageal cancer have a high rate of adverse perioperative complications and associated longer hospital stay.^11^, ^12^ As an emerging surgical approach, minimally invasive esophagectomy (MIE) is designed to improve poor outcomes compared to traditional open esophagectomy (OE). Several previous studies have demonstrated that MIE could reduce estimated blood loss (EBL) and postoperative complications, shorten the length of hospital stay, and have similar early oncologic outcomes.^13^, ^14^ Up to now, little work is being done exploring the effect of elevated body mass index (BMI) on perioperative and oncological outcomes in elderly patients treated with MIE. In the current study, by reviewing a large sample of prospectively collected data, we attempted to evaluate the association between elevated BMI and perioperative and oncological outcomes in MIE.

## 2 MATERIALS AND METHODS

### 2.1 Study design and patients

**Figure 1:**
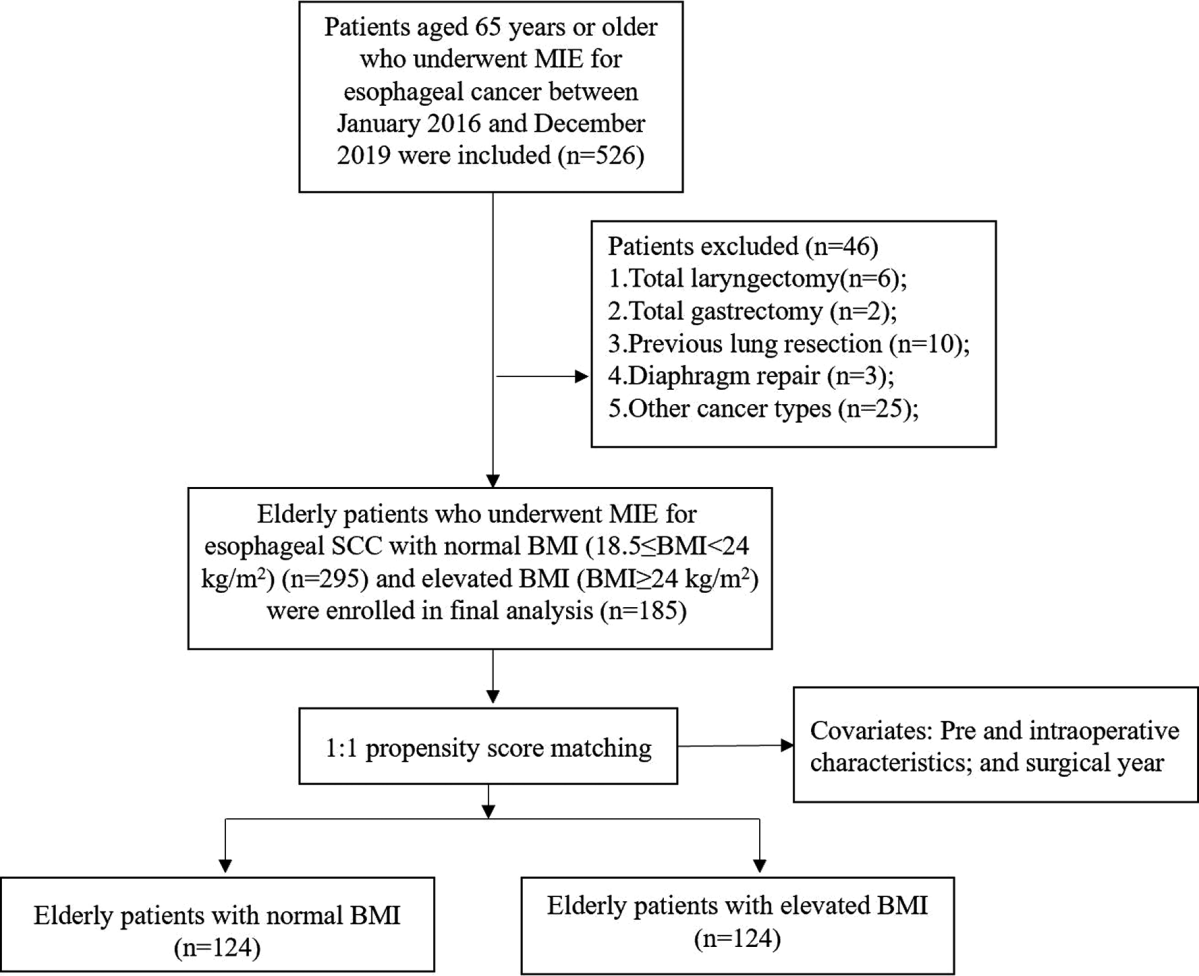
Patient flowchart

From January 2016 to December 2019, we performed a monocentric retrospective analysis based on a prospectively collected database, including 526 consecutive patients aged 65 years or older who underwent minimally invasive McKeown esophagectomy (MIE) for esophageal squamous cell carcinoma (SCC). Excluded patients were listed in the flow diagram (Figure 1). A total of 480 elderly patients were eventually enrolled. The Ethics Committee of Shanghai Chest Hospital approved this study (IS21121) and waived the need for informed consent.

### 2.2 Anesthesia protocol

All patients were routinely monitored by electrocardiogram, pulse oximetry, and capnography. Catheterization of the radial artery and right internal jugular central venous were used to monitor invasive blood pressure (IBP). Patients received lung‐protective ventilation (LPV) strategies, including low‐tide ventilation based on ideal body weight (≤8 mL/kg), PEEP = 5 cmH_2_O, lung recruitment, and maintenance of airway pressure < 30 cmH_2_O, and were located in the lateral position required for surgery. After the operation, all patients received patient‐controlled analgesia (PCA) pump, including sufentanil1.0 μg/kg + desoxocin 0.4 mg/kg.

### 2.3 Technique of operation

All patients underwent robot‐assisted MIE (RAMIE) or thoracoscopic‐assisted MIE (TAMIE) with two‐ or three‐field lymphadenectomy by the same group of thoracic surgeons. The procedure of MIE involved the thoracoscopic movement of the esophagus followed by laparoscopic or laparotomy to establish a gastric tube and cervical esophagogastric anastomosis. The choice of the two approaches was completely based on the chief surgeon's preference after consideration of preoperative evaluation, operative planning, patient benefit, and surgical experience. Patients received either hand‐sewn or circular anastomosis esophagogastric anastomosis in this study.

### 2.4 Data collection

Perioperative data were prospectively pooled from our medical record system, including patient's baseline and intraoperative characteristics, intra‐and postoperative complications regarding hypoxemia, pulmonary complications, surgical complications (EBL, transfusion, new‐onset arrhythmia, conversion to thoracotomy, anastomotic leakage, chylothorax, and reoperation), intensive care unit (ICU) and 30‐day readmissions, the length of hospital stay (LOS), and oncological outcomes (R0 dissection and the number of dissected lymph nodes).

### 2.5 Definition

Postoperative pulmonary complications (PPCs) were defined based on the European Perioperative Clinical Outcomes (EPCO),^15^ including atelectasis, pulmonary infection, and respiratory failure. Perioperative new‐onset arrhythmia included incidents of atrial fibrillation (AF) and atrial flutter based on the 2014 Guidelines of the American Association of Thoracic Surgeons (AATS).^16^ By referring to the Guidelines for Prevention and Control of Overweight and Obesity in Chinese Adults,^17^ two groups were stratified by BMI status: normal (18.5 ≤ BMI < 24 kg/m^2^) and elevated groups (BMI ≥ 24 kg/m^2^).

### 2.6 Statistical analysis

Continuous variables were compared between normal and elevated groups using Two independent sample t‐test or Mann–Whitney U test. Chi‐square test or Fisher exact test, depending on the sample size, were used to compare categorical variables. A 1:1 propensity score matching (PSM)^18^ analysis with a caliper size of 0.05 was used to lessen the selection bias and other potential confounding effects. All pre‐, intraoperative variables, and surgical years were included in the PSM. Standardized mean difference (SMD) between two cohorts on all covariables before and after matching was calculated, with differences of <10% indicating adequate balance in the cohort. Statistical analysis was conducted using the SPSS 26.0 software (IBM Corp.). R version 4.1.2 was used with the tableone, ggplot2, reshape2, survey, and Matching packages. A *p* value <0.05 was statistically significant.

## 3 RESULTS

### 3.1 Study cohort

**Figure 2:**
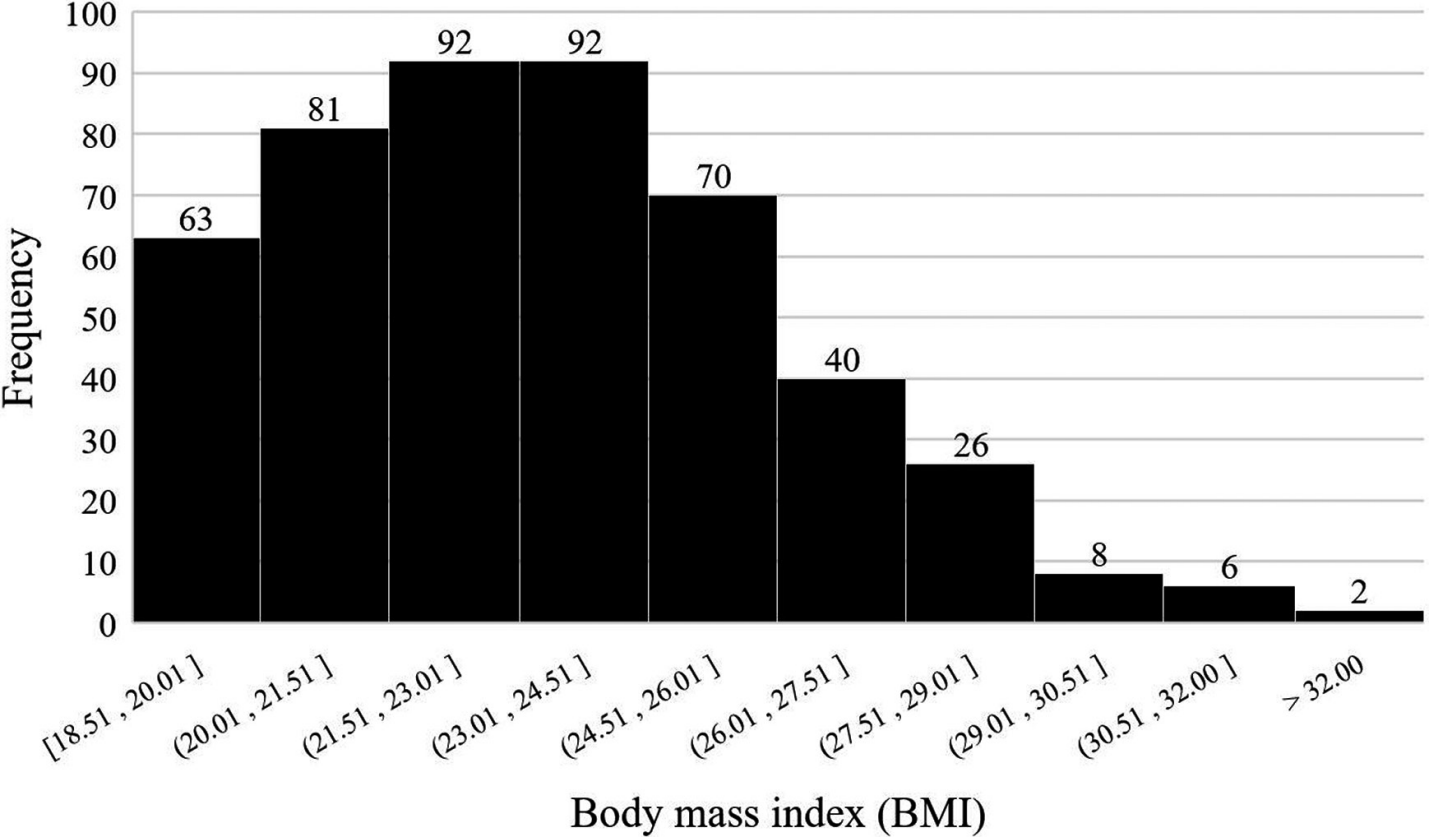
Body mass index distribution

From January 2016 to December 2019, 480 elderly patients with a mean age of 70.2 years (range: 65–87) underwent MIE for esophageal SCC, of which 42.1% (202 out of 480) underwent RAMIE and 57.9% (278 out of 480) underwent TAMIE, and 38.5% (185 out of 480) were eligible for elevated BMI, with a mean BMI of 26.3 ± 1.9 kg/m^2^ (Figure 1). The BMI distribution of all enrolled patients were depicted in Figure 2.

Patients with elevated BMI had a higher incidence of hypertension (24.9% vs. 14.9%, *p* = 0.007) and a lower incidence of chemoradiotherapy (8.1% vs. 15.6%, *p* = 0.017), and better preoperative pulmonary function (FEV_1_/FVC, 101.0 ± 10.8 vs. 98.5 ± 11.0, *p* = 0.016; DLCO%, 98.2 ± 22.0 vs. 94.4 **±** 19.8, *p* **=** 0.054) when compared with their counterparts (Table 1). Additionally, patients with elevated BMI required prolonged operative time (278.9 ± 62.7 vs. 261.7 ± 57.2 mins, *p* = 0.002) compared with those with normal BMI (Table 2).

**Table 1:** Baseline characteristics stratified by BMI

Variables^a^	18.5 ≤ BMI < 24 kg/m^2^ (*n* = 295)	BMI ≥ 24 kg/m^2^ (*n* = 185)	SMD	*p* value
Age, years	70.2 ± 4.4	70.3 ± 4.1	0.032	0.733
Sex			0.080	0.398
Male sex	228 (77.3)	149 (80.5)		
Female sex	67 (22.7)	36 (19.5)		
ASA grade			0.005	0.960
I	1 (0.3)	1 (0.5)		
II	214 (72.5)	133 (71.9)		
III/IV	80 (27.1)	51 (27.6)		
Comorbidity				
Hypertension	44 (14.9)	46 (24.9)	0.251	0.007^b^
Diabetes mellitus	16 (5.4)	8 (4.3)	0.051	0.591
Stroke or TIA	3 (1.0)	3 (1.6)	0.053	0.680
Coronary artery disease	4 (1.4)	1 (0.5)	0.084	0.653
FEV_1_/FVC, %	98.5 ± 11.0	101.0 ± 10.8	0.228	0.016^b^
DLCO%	94.4 ± 19.8	98.2 ± 22.0	0.179	0.054
Tumor size, cm	3.3 ± 1.5	3.5 ± 1.5	0.173	0.066
Chemoradiotherapy	46 (15.6)	15 (8.1)	0.233	0.017^b^
Type of anesthesia			0.031	0.740
GA alone	247 (83.7)	157 (84.9)		
GA plus TPVB	48 (16.3)	28 (15.1)		
pT stage			0.056	0.484
T1	66 (22.4)	41 (22.2)		
T2	85 (28.8)	48 (25.9)		
T3	143 (48.5)	93 (50.3)		
T4a	1 (0.3)	3 (1.6)		

Abbreviations: BMI: Body mass index; SMD: Standardized mean difference; ASA: American Society of Anesthesiology; TIA: Transient cerebral ischemic attack; FEV_1_: Forced expiratory volume in 1 s; FVC: Forced vital capacity; DLCO: Diffusion capacity for carbon monoxide; GA‐TPVB: General anesthesia combined with the thoracic paravertebral blockade.

^a^ Continuous data are shown as mean ± standard deviation and categoric data as number (%).

^b^ Statistically significant (*p* < 0.05).

**Table 2:** Intraoperative characteristics stratified by BMI

Variables^a^	18.5 ≤ BMI < 24 kg/m^2^ (*n* = 295)	BMI ≥ 24 kg/m^2^ (*n* = 185)	SMD	*p* value
Colloidal fluid volume, mL	1000 (500–1000)	1000 (500–1000)	0.087	0.429
Crystal fluid volume, mL	1000 (1000–1500)	1000 (1000–1500)	0.113	0.260
Total fluid volume, mL	2000 (1500–2500)	2000 (2000–2500)	0.138	0.182
Operative time, mins	261.7 ± 57.2	278.9 ± 62.7	0.287	0.002^b^
Clinical nodal involvement	134 (45.4)	91 (49.2)	0.075	0.421
Approach			0.003	0.978
VATS	171 (58.0)	107 (57.8)		
RATS	124 (42.0)	78 (42.2)		
Type of anastomosis			0.103	0.258
Hand‐sewn	11 (3.7)	11 (5.9)		
Circular stapled	284 (96.3)	174 (94.1)		
Lymphadenectomy			<0.001	0.998
Two‐field	244 (82.7)	153 (82.7)		
Three‐field	51 (17.3)	32 (17.3)		
Location of resection			0.007	0.941
Upper thoracic	36 (12.2)	23 (12.4)		
Middle‐lower thoracic	259 (87.8)	162 (87.6)		
Surgical procedure			0.129	0.176
Thoracoscopic‐laparotomy	41 (13.9)	18 (9.7)		
Thoracoscopic‐laparoscopy	254 (86.1)	167 (90.3)		
Postoperative ICU admission	229 (77.6)	151 (81.6)	0.099	0.294

Abbreviations: BMI: Body mass index; SMD: Standardized mean difference; VATS: Video‐assisted thoracoscopic surgery; RATS: Robotic‐assisted thoracoscopic surgery; ICU: Intensive care unit.

^a^ Continuous data are shown as mean ± standard deviation or as median (interquartile range) and categoric data as number (%).

^b^ Statistically significant (*p* < 0.05).

### 3.2 **Perioperative and oncological outcomes** before and after a 1:1 PSM

Compared with normal‐BMI patients, the rate of intraoperative hypoxemia (12.2% vs. 21.6%, *p* = 0.006) was higher in patients with elevated BMI (Table 3). The differences in intraoperative EBL, transfusion, new‐onset arrhythmia and conversion rate, and postoperative outcomes regarding pulmonary and surgical complications, ICU and 30‐day readmissions, LOS and oncological outcomes including R0 dissection and the number of dissected lymph nodes between two groups were comparable (Table 3).

**Table 3:** Perioperative complications and oncological outcomes stratified by BMI

Variables^a^	18.5 ≤ BMI < 24 kg/m (*n* = 295)	BMI ≥ 24 kg/m^2^ (*n* = 185)	*p* value
Intraoperative complications			
Hypoxemia	36 (12.2)	40 (21.6)	0.006^b^
Estimated blood loss, mL	200 (200–200)	200 (200–200)	0.456
Transfusion	12 (4.1)	5 (2.7)	0.431
New‐onset arrhythmia	13 (4.4)	14 (7.6)	0.199
Conversion to thoracotomy	3 (1.0)	5 (2.7)	0.270
Postoperative complications			
PPCs	149 (50.5)	87 (47.0)	0.458
Atelectasis	23 (7.8)	8 (4.3)	0.132
Pulmonary infection	138 (46.8)	83 (44.9)	0.682
Respiratory failure	19 (6.4)	15 (8.1)	0.488
Surgical complications			
New‐onset arrhythmia	17 (5.8)	6 (3.2)	0.208
Transfusion	38 (12.9)	27 (14.6)	0.593
Anastomotic leakage	15 (5.1)	9 (4.9)	0.914
Chylothorax	14 (4.7)	4 (2.2)	0.147
Reoperation	6 (2.0)	4 (2.2)	1.000
ICU readmission	15 (5.1)	9 (4.9)	0.914
30‐day readmission	6 (2.0)	4 (2.2)	1.000
Length of hospital stay, day	9 (7–11)	9 (7–11)	0.422
Oncological outcomes			
R0 dissection	272 (92.2)	173 (93.5)	0.591
Number of dissected LN	20 (15–27)	20 (14–28)	0.250

Abbreviations: BMI: Body mass index; PPCs: Postoperative pulmonary complications; ICU: Intensive care unit; LN: lymph nodes.

^a^ Continuous data are shown as mean ± standard deviation or median (interquartile range) and categoric data as number (%).

^b^ Statistically significant (*p* < 0.05).

All patients’ baseline, intraoperative characteristics, and surgical year were comparable among two cohorts after a 1:1 PSM (Table 4, 5). We investigated outcomes in 248 patients (124 pairs), the difference in both intra‐ and postoperative complications between the two cohorts was not significant (Table 6). Also, there was no significant difference between two cohorts in terms of R0 dissection (vs. normal‐BMI; 88.7% vs. 93.5%, *p* = 0.180) and the number of dissected lymph nodes (vs. normal‐BMI; median (interquartile range); 20 (16–28) vs. 20 (14–28), *p* = 0.373) (Table 6).

**Table 4:** Baseline characteristics stratified by BMI after a 1:1 PSM

Variables^a^	18.5 ≤ BMI < 24 kg/m^2^ (*n* = 124)	BMI ≥ 24 kg/m^2^ (*n* = 124)	SMD	*p* value
Age, years	70.4 ± 4.4	70.2 ± 4.1	0.045	0.492
Sex			0.063	0.621
Male sex	103 (83.1)	100 (80.6)		
Female sex	21 (16.9)	24 (19.4)		
ASA grade			0.069	0.835
I	1 (0.8)	1 (0.8)		
II	86 (69.4)	90 (72.6)		
III/IV	37 (29.8)	33 (26.6)		
Comorbidity				
Hypertension	23 (18.5)	22 (17.7)	0.021	0.869
Diabetes mellitus	5 (4.0)	6 (4.8)	0.039	0.758
Stroke or TIA	2 (1.6)	1 (0.8)	0.074	1.000
Coronary artery disease	0 (0)	1 (0.8)	0.007	1.000
FEV_1_/FVC, %	99.4 ± 9.8	99.6 ± 10.4	0.023	0.856
DLCO%	94.7 ± 20.4	96.9 ± 21.2	0.010	0.414
Tumor size, cm	3.3 ± 1.5	3.4 ± 1.4	0.012	0.424
Chemoradiotherapy	14 (11.3)	12 (9.7)	0.052	0.678
Type of anesthesia			0.088	0.191
GA alone	97 (78.2)	105 (84.7)		
GA plus TPVB	27 (21.8)	19 (15.3)		
pT stage			0.038	0.438
T1	30 (24.2)	29 (23.4)		
T2	28 (22.6)	29 (23.4)		
T3	66 (53.2)	63 (50.8)		
T4a	0 (0)	3 (2.4)		

Abbreviations: BMI: Body mass index; PSM: Propensity score matching; SMD: Standardized mean difference; ASA: American Society of Anesthesiology; TIA: Transient cerebral ischemic attack; FEV_1_: Forced expiratory volume in 1 s; FVC: Forced vital capacity; DLCO: Diffusion capacity for carbon monoxide; GA‐TPVB: General anesthesia combined with thoracic paravertebral blockade.

^a^ Continuous data are shown as mean ± standard deviation and categoric data as number (%).

^b^ Statistically significant (*p* < 0.05).

**Table 5:** Intraoperative characteristics stratified by BMI after a 1:1 PSM

Variables^a^	18.5 ≤ BMI < 24 kg/m^2^ (*n* = 124)	BMI ≥ 24 kg/m^2^ (*n* = 124)	SMD	*p* value
Colloidal fluid volume, mL	1000 (500–1000)	1000 (500–1000)	0.089	0.106
Crystal fluid volume, mL	1000 (1000–1500)	1000 (1000–1500)	0.003	0.933
Total fluid volume, mL	2000 (2000–2500)	2000 (1700–2500)	0.077	0.215
Operative time, mins	267.0 ± 57.0	272.2 ± 62.4	0.087	0.492
Clinical nodal involvement	60 (48.4)	65 (52.4)	0.080	0.607
Approach			0.065	
VATS	74 (59.7)	70 (56.5)		
RATS	50 (40.3)	54 (43.5)		
Type of anastomosis			0.068	0.355
Hand‐sewn	4 (3.2)	7 (5.6)		
Circular stapled	120 (96.8)	117 (94.4)		
Lymphadenectomy			0.043	0.735
Two‐field	102 (82.3)	104 (83.9)		
Three‐field	22 (17.7)	20 (16.1)		
Location of resection			0.069	0.584
Upper thoracic	16 (12.9)	19 (15.3)		
Middle‐lower thoracic	108 (87.1)	105 (84.7)		
Surgical procedure			0.089	0.162
Thoracoscopic‐laparotomy	7 (5.6)	13 (10.5)		
Thoracoscopic‐laparoscopy	117 (94.4)	111 (89.5)		
Postoperative ICU admission	93 (75.0)	96 (77.4)	0.057	0.655

Abbreviations: BMI: Body mass index; PSM: Propensity score matching; SMD: Standardized mean difference; VATS: Video‐assisted thoracoscopic surgery; RATS: Robotic‐assisted thoracoscopic surgery; ICU: Intensive care unit.

^a^ Continuous data are shown as mean ± standard deviation or as median (interquartile range) and categoric data as number (%).

^b^ Statistically significant (*p* < 0.05).

**Table 6:** Perioperative complications and oncological outcomes stratified by BMI after a 1:1 PSM

Variables^a^	18.5 ≤ BMI < 24 kg/m^2^ (*n* = 124)	BMI ≥ 24 kg/m^2^ (*n* = 124)	*p* value
Intraoperative complications			
Hypoxemia	18 (14.5)	27 (21.8)	0.138
Estimated blood loss, mL	200 (200–200)	200 (200–200)	0.447
Transfusion	3 (2.4)	5 (4.0)	0.722
New‐onset arrhythmia	5 (4.0)	11 (8.9)	0.121
Conversion to thoracotomy	0 (0)	3 (2.4)	0.247
Postoperative complications			
PPCs	54 (43.5)	57 (46.0)	0.702
Atelectasis	8 (6.5)	7 (5.6)	0.790
Pulmonary infection	49 (39.5)	54 (43.5)	0.519
Respiratory failure	7 (5.6)	8 (6.5)	0.790
Surgical complications			
New‐onset arrhythmia	6 (4.8)	3 (2.4)	0.500
Transfusion	10 (8.1)	19 (15.3)	0.075
Anastomotic leakage	6 (4.8)	6 (4.8)	1.000
Chylothorax	6 (4.8)	4 (3.2)	0.519
Reoperation	2 (1.6)	4 (3.2)	0.684
ICU readmission	3 (2.4)	5 (4.0)	0.722
30‐day readmission	3 (2.4)	4 (3.2)	1.000
Length of hospital stay, day	8 (7–10)	9 (7–11)	0.101
Oncological outcomes			
R0 dissection	110 (88.7)	116 (93.5)	0.180
Number of dissected LN	20 (16–28)	20 (14–28)	0.373

Abbreviations: BMI: Body mass index; PSM: Propensity score matching; PPCs: Postoperative pulmonary complications; ICU: Intensive care unit; LN: lymph nodes.

^a^ Continuous data are shown as mean ± standard deviation or median (interquartile range) and categoric data as number (%).

^b^ Statistically significant (*p* < 0.05).

## 4 DISCUSSION

A total of 185 elderly patients who underwent MIE for SCC were eligible for elevated BMI. This study found that elderly patients with elevated BMI had similar rates of perioperative complications and comparable oncological outcomes compared to patients with normal BMI. Thus, elevated BMI in elderly patients should not be a hindrance to preoperative evaluation, risk stratification, and surgical planning during MIE.

The World Health Organization has recommended BMI thresholds for underweight (<18.5 kg/m^2^), normal weight (18.5–24.9 kg/m^2^), overweight (25–29.9 kg/m^2^), and obesity (>30 kg/m^2^) to predict risk for all cancer types and non‐cancer diseases. However, whether the above criteria applied to Asian populations remains controversial.^17^, ^19^, ^20^ The elevated BMI classification in this investigation by referring to the Guidelines for Prevention and Control of Overweight and Obesity in Chinese Adults, which may be more suitable for the Chinese population.^17^

MIE has been established to improve perioperative poor outcomes with regard to the standard open approaches.^13^, ^14^ Presumably, the proportion of elderly patients with elevated BMI undergoing MIE will constantly increase in the future.^6^ Intuitively, it seems that preoperative comorbidities related to elevated BMI, impaired pulmonary function, prolonged operative time, tissue fragility and reduced mobility should be associated with an increased risk of complications among elderly patients. Therefore, it is mandatory to fully understand the impact of elevated BMI on perioperative outcomes in elderly patients.

In terms of intraoperative complications, before a 1:1 PSM, patients with elevated BMI had a high rate of hypoxemia and prolonged operative time and developed comparable EBL, transfusion, new‐onset arrhythmia, and conversion rates compared to normal‐BMI patients, but none of these difference**s** were significant after matching. Our previously published literature echoed these results and showed that elevated BMI was not associated with high rates of intraoperative conversions and new‐onset arrhythmia.^21^, ^22^ Similarly, Salem^23^ and Kilic^24^ have evaluated the impact of BMI on perioperative clinical outcomes after MIE and open esophagectomy, respectively, and found that elevated BMI was associated with longer operative time but not with a significant increase in EBL, despite differences in baseline characteristics among these studies.

In this study, no difference was observed between patients with normal and elevated BMI for pulmonary and surgical complications, ICU and 30‐day readmissions, and LOS. Several other studies also showed no higher incidence of complications following esophagectomy with increased BMI.^23^, ^24^, ^25^, ^26^ An analysis of the Nationwide Inpatient Database in Japan demonstrated that BMI showed U‐shaped dose‐response associations with mortality, major complications, and multiple complications.^27^ Conversely, using the Society of Thoracic Surgeons (STS) General Thoracic Surgery Database, Mitzman et al conducted a retrospective study and concluded that overweight and obese I was associated with decreased risk for most complication types.^28^

Our investigation also assessed the effects of elevated BMI on oncological outcomes regarding R0 dissection and the number of dissected lymph nodes after MIE in elderly patients and showed that the difference was not significant between patients with normal and elevated BMI. And the results were consistent with other published studies.^26^, ^29^ Besides, Sachdeva and colleagues’ research using STS General Thoracic Surgery Database also indicated that R0 resection or lymphadenectomy did not differ among five BMI groups.^30^ Given the consistency of these findings, we tend to believe that neither older age nor elevated BMI may be independent factors affecting oncological outcomes.

Potential defects of our study include as follows. First, as a retrospective study based on a prospectively collected database, it has inherent design biases. Besides, this study did not further subdivide elevated BMI into overweight and obesity, as only 30 patients enrolled were considered obese. Second, due to the limited granularity of postoperative care data, some poor outcomes such as pain control and other surgical complications could not be pooled in this study. Third, the relationship between elevated BMI and long‐term prognosis following MIE in elderly patients needs further investigation.

## 5 CONCLUSIONS

By performing a single‐center retrospective study of 480 elderly patients receiving MIE for esophageal SCC, our study found that elevated BMI did not increase perioperative adverse complications and oncological outcomes. These data contribute to the increasing body of evidence that elevated BMI in older patients should not exclude candidates for MIE for esophageal SCC.

## CONFLICT OF INTEREST

All authors have no conflicts of interest to declare.

## AUTHOR CONTRIBUTION

Chaoyang Tong and Huijie Lu: study conception, design, acquisition of data, and drafting of the manuscript. Chaoyang Tong, Jingxiang Wu, and Hongwei Zhu: analysis and interpretation of data. Jingxiang Wu and Hongwei Zhu: critical revision. All authors contributed to the article and approved the submitted version.

## ETHICS STATEMENT

The Ethics Committee of Shanghai Chest Hospital approved the study (IS21121) and waived the need for informed consent.

## Data Availability

Our research team could provide original data under reasonable request and with permission from Shanghai Chest Hospital.
